# Work stress on rise? Comparative analysis of trends in work stressors using the European working conditions survey

**DOI:** 10.1007/s00420-020-01593-8

**Published:** 2020-11-01

**Authors:** M. Rigó, N. Dragano, M. Wahrendorf, J. Siegrist, T. Lunau

**Affiliations:** 1 Institute of Medical Sociology, Centre for Health and Society, Medical Faculty, University of Düsseldorf, Düsseldorf, Germany; 2 Senior Professorship on Work Stress Research, Medical Faculty, University of Düsseldorf, Düsseldorf, Germany

**Keywords:** Work stressors, Effort-reward imbalance, Job strain, Trends, Cross-national study, Occupational disparities

## Abstract

**Objective:**

The rapid transformation of labor markets has been accompanied by the belief of rising stress at work. However, empirical evidence on such trends based on reliable survey data is scarce. This study analyzes long-term trends in well-established measures of work stressors across Europe, as well as potential occupational differences.

**Methods:**

We use repeated cross-sectional data of 15 European countries from waves 1995, 2000, 2005, 2010, and 2015 of the European Working Conditions Surveys. We apply three-way multilevel regressions (with employees nested in country-years, which are in turn nested in countries) to analyze trends in work stressors measured according to the demand-control and effort-reward imbalance models. Trends by occupational groups are also assessed.

**Results:**

Our findings suggest that work stress generally increased from 1995 to 2015, and that the increase was mostly driven by psychological demands. People working in lower-skilled occupations had generally higher levels of job strain and effort-reward imbalance, as well as they tend to have a steeper increase in job strain than people working in higher-skilled occupations. Most of the change occurred from 1995 to 2005.

**Conclusion:**

Our results indicate that work stress has been on rise since 1995, specifically for people working in disadvantageous occupations. This directs the attention to the vulnerable position of the least skilled and also to the use of preventive measures to counteract some of the disadvantages experienced by this occupational group.

## Introduction

The detrimental health consequences of work stress have been widely documented in the occupational health research. Long-term exposure to adverse psychosocial working conditions have been found to increase the chance of developing depressive disorders (Rugulies et al. 2017; Madsen et al. 2017; Theorell et al. 2015), cardiovascular diseases (Kivimäki et al. 2012; Dragano et al. 2017) or musculoskeletal diseases (Lang et al. 2012). Additionally, work stress was found to be associated with lower employee productivity (Burton et al. 1999), higher rate of sickness absence (Götz et al. 2018; Mortensen et al. 2017) and an earlier exit from the labor force (Hintsa et al. 2015; Juvani et al. 2014; Mäcken 2019).

Despite the wide range of evidence on the negative consequences of work stress and its policy relevance, only a small number of studies analyze how the prevalence of work stressors has changed during the last decades. Labor markets have been undergoing profound structural changes. Globalization, innovations in technology, digitalization lead to new forms of work organizations and changing working conditions. The rapid transformation of labor markets has been accompanied by the belief that work stress is rising. However, little is known about long-term trends in work stressors based on large-scale survey data. Our paper takes an approach to contribute to the literature by analyzing the long-term evolution of the prevalence of distinct work stressors with relevance to health.

Available studies analyzing trends in EU countries documented no clear pattern. One prevailing trend is the increase in work intensity from 1995 to 2005 (Greenan et al. 2014; Lopes et al. 2014). Lopes et al. (2014) also found a decrease in work autonomy between 1995 and 2010. Malard et al. (2013) examining short-term trends found that skill discretion, decision latitude and job insecurity deteriorated, while job promotion and work-life balance improved from 2005 to 2010. However, a shortcoming of these studies is that they analyze a limited time span and often rely on measures of work stress, which have not been linked to health outcomes previously. Additionally, there are substantial differences in statistical approaches often restricting the analysis to the descriptive evaluation of trends. Therefore, results are difficult to compare and do not allow a more comprehensive contribution to the literature. Our study is the first that uses five waves of the EWCS and analyzes trends in work stressors over a 20-year period relying on well-known theoretical concepts and applying up-to-date statistical modelling. Previous studies using similar operationalization of work stress focused only on two waves (Malard et al. 2013) or used more waves but relied on a different conceptualization of work stress (e.g. Greenan et al. 2014; Lopes et al. 2014).

Our main measures of work stress are job strain relying on the demand-control model (Karasek and Theorell 1990) and effort-reward imbalance (ERI) based on the effort-reward imbalance model (Siegrist 1996). Though the complete lists of original survey items are not available in the EWCS, we created proxy measures as close as possible to the original constructs. In the framework of the demand-control model work-related stress is the result of high psychological job demands and the lack of control available to employees to perform their tasks. The effort-reward imbalance model characterizes stressful jobs by the combination of high effort and low reward where rewards include money, esteem, career opportunities and job security. These measures of work stress have been validated using a number of studies from a wide range of countries (Siegrist et al. 2014) and have been successively shown to be related to the incidence of numerous diseases (e.g. Kivimäki et al. 2012; Rugulies et al. 2017; Lang et al. 2012). Evidence on the close correspondence between the original constructs and their proxy or partial versions have been also provided (Fransson et al. 2012; Karasek et al. 2007). Though our paper focuses on trends in work stress measures, we also analyze trends in the underlying constructs. For job strain, trends in psychological demands and control, for ERI, trends in effort and reward will be also evaluated.

In addition to strengthening the evidence on these trends, it is also important to explore whether differences between occupational groups exist. Previous research has documented that employees in lower occupational groups reported higher levels of work stress compared to employees in higher occupational position (Wahrendorf et al. 2012; Brunner et al. 2004). This can be partly explained by occupational characteristics. Lower-level occupations tend to be characterized by lower levels of control (e.g. job autonomy) and lower rewards (e.g. job security) (Parent-Thirion et al. 2015). We also assume that the trend differs between occupational groups. Global labor market developments and long-term trends in institutional developments may have a differing impact on employees with different skill and occupational backgrounds (Eurofound 2013). For instance, new technologies disfavoring routine work increased the demand for employees at the bottom and upper extreme of the skill distribution, a process described in the literature as labour market polarization (Goos et al. 2014). Additional theories suggest that institutional changes of the labour market such as labour market deregulation or the position of labour unions could also have an impact on employment and wage opportunities along the skill distribution (Koeniger et al. 2007; DiNardo et al. 1996). Furthermore, educational upgrading, shift from routine tasks to analytical activities, increase in the demand of services, demographic ageing processes, a rising share of immigrants and their relatively high share in elementary occupations, increasing global trade and the offshoring of certain tasks could also have an influence on the job opportunities, wages and working conditions of employees in different occupations (Jensen et al. 2019; Eurofound 2013). Numerous studies provide evidence on how wages and employment opportunities evolved among employees with different skill levels (Goos et al. 2014; Fernández-Macías 2012). However, we know little about how other aspects of working conditions, in particular experienced work stress, have changed in those occupations. The few existing studies indicate that low-skilled workers are often more affected by the deterioration of working conditions (Lopes et al. 2014; Malard et al. 2013). However, so far only limited evidence on shorter time periods and using alternative measures of adverse working conditions exist. Therefore, more studies are needed to enhance our understanding of this research question.

Our analysis drawing upon well-established measures of work stress can have a unique contribution as these measures have been previously linked to health outcomes. Our study helps to explore whether the labor market changes that have occurred during the last 20–25 years have led to an increasing or decreasing amount of work stress, and whether all groups of workers have been affected to a similar extent.

We use data from the European Working Conditions Survey comprising 15 countries from 5 waves over a time period of 20 years. Our work investigates the following research questions: (1) Did the level of work stress change during the last 20 years? (2) If so, did these trends differ by occupational groups?

Exploring answers to the above questions has both scientific and policy relevance. From a scientific point of view, it might improve our understanding of the impacts of changes in global labor markets on working conditions. The consequences of global changes have been intensively studied in economics but remain a relatively understudied field in occupational health research. Second, our work may also contribute to explaining health inequalities as long as the distributions of working conditions and health consequences are unequal between employees with different occupations. From a policy point of view, our results can highlight the necessity of policy actions that help to buffer some of the negative consequences of global changes experienced disproportionately by certain demographic groups. One aim of our work is to identify those groups, and as such, findings may offer important inputs to policy design at national level.

## Methods

### Data

We use data from the European Working Conditions Survey (EWCS). The EWCS is carried out by Eurofound in every 5 years since 1990 and provide detailed information on employees’ working conditions and demographic characteristics. The first wave is considered to be a prototype including only 12 countries. Therefore, in our analysis we decided to include the five waves starting from 1995. These waves include a comparable set of survey questions and countries. Job strain could be computed in all five waves, and ERI in the last three waves. Employees are surveyed cross-sectionally in each wave. However, the EWCS has a longitudinal character as countries are sampled in multiple waves. Therefore, the dataset includes non-repeated observations from a large random sample of individuals and repeated observations from European countries. The country sample sizes are around 1000 in each wave with a few exceptions where around 2000 employees are interviewed. The sampling method followed a multistage, stratified and clustered design. Our trend analysis focuses on the 15 countries included in the sample in all five waves: Belgium, Denmark, Germany, Greece, Spain, France, Ireland, Italy, Luxembourg, Netherlands, Austria, Portugal, Finland, Sweden and UK. We excluded from the sample those aged below 15 and above 65 because they may have particular work situations (e.g. work after pension age). For similar reasons, we also excluded persons working <8 h per week (1284) and those who were self-employed (12,935). The resulting database for our analysis differed by measures of work stress used (see below for measurement details) and included 74,959 observations in case of job strain and 45,399 observations in case of ERI.

### Variables, measurement

#### Working conditions

Our analysis focuses on theory-based work stress constructs, which have been previously linked to health outcomes. Relying on the available survey items of the EWCS, proxies for job strain based on the demand-control (Karasek and Theorell 1990) and ERI based on the effort-reward imbalance model (Siegrist 1996) were computed. Job strain could be calculated using all fives waves from 1995, while the measurement for ERI was only possible for the waves 2005, 2010, and 2015. The exhaustive list of underlying survey items and corresponding composite constructs are summarized by Table 4 in the Appendix.

Though previous literature often defines work stressors as dummy variables indicating high or low levels (Niedhammer et al. 2012), we specify them as continuous variables. In this way, we could utilize all available information, which might provide more accurate analysis on trends. Similar approach was taken by Malard et al. (2013) and Myers et al. (2019). Job strain is defined as the ratio of psychological demands and control with higher values indicating higher job strain. Psychological demands were constructed using two single survey items referring to work intensity (Cronbach’s alpha 0.72). Control is defined as the mean of skill discretion and decision authority. Skill discretion is created using four single items characterizing job variety and job complexity (Cronbach’s alpha 0.41). Decision authority uses three items describing the flexibility of the employee in terms of order, method and speed of the tasks (Cronbach’s alpha 0.76). Items were coded so that higher values indicate higher levels of demand, skill discretion or decision authority. Following previous literature (Niedhammer et al. 2012), responses to all single items have been standardized to have a range between 1 and 2. The composite variables of skill discretion and decision authority have been defined as the mean of the single items. As such, all single items and the composite constructs of psychological demands, skill discretion and decision authority have a range between 1 and 2, and job strain lies between 0.5 and 2.

Effort-reward imbalance (ERI) is defined as the ratio of effort and reward with higher values indicating higher work stress. Effort is defined identically to psychological demands. Reward is composed of five survey items referring to job security, job promotion prospects, esteem reward, financial reward and social support from colleagues and manager (Cronbach’s alpha 0.47). Again, items are defined in a way so that higher values indicate higher levels of effort and reward and they have been standardized to lie between the range of 1 and 2. Thus, ERI, similarly to job strain, ranges between 0.5 and 2.

#### Other covariates

Our regressions include the following explanatory variables to control for the different composition of countries: age (defined by group dummies for employees below 30, between 30 and 50 and over 50 years old), gender (male, female), contract (indefinite contract, fixed term contract, temporary agency employment, apprenticeship or other), industry (5 groups by NACE categories) and occupational position. These variables may be correlated with employees’ perceptions of work stress (Niedhammer et al. 2012). Most of the covariates have been commonly included in previous papers as well (Malard et al. 2013; Greenan et al. 2014). The measurement of occupational position is based on the ISCO-88 classification (International Standard Classification of Occupations) developed by the International Labour Office. ISCO codes facilitate the international comparison of occupational statistics. ISCO classifies jobs into 390 different categories, which are grouped into 10 major groups and four broad hierarchical groups. For our analyses, we use the four broad groups, which are defined by the skill level of the tasks: Skill level 4—high skilled clerical (HC, based on ISCO major groups 1, 2 and 3); Skill level 3—low skilled clerical (LC, based on ISCO major groups 4 and 5); Skill level 2—high skilled manual (HM, based on ISCO major groups 6 and 7); Skill level 1—low skilled manual (LM, based on ISCO major groups 8 and 9). Armed forces (ISCO major group 0) are excluded from the analysis.

### Statistical analyses

Our analytical strategy relies on multilevel regressions where work stressors are regressed on a set of covariates. The modeling takes into account the three-level hierarchical structure of the EWCS with employees (level 1) nested within country-years (level 2; countries are observed in several consecutive waves) nested within countries (level 3). Multilevel models explicitly assume that the error term has the same known hierarchical structure as the dataset. In our case, the error term is partitioned into a country, a country-year and an individual component each having the usual assumptions of independence, normality and homoscedasticity. As such, compared to traditional OLS methods treating each units of observations as independent, multilevel models generate the correct standard error and avoid the downward bias that would have been created if OLS was applied.

Our main control variables are the wave dummies, which allow for a flexible modeling of time. Besides, as specified in the previous section, controls to adjust for compositional differences between the countries have been included.

To address the question whether time trends differ by occupational groups, we added a product term composed of occupational dummies and wave dummies. Wald test is used to test the joint significance of the interaction terms. As the estimated coefficients on the interaction terms are not straightforward to interpret, we only present the predicted values of work stressors by occupation. Besides, average marginal effects (AME) are computed, assessing whether the wave-to-wave changes in work stressors, or, occupational differences within one wave are significant.

Some of the specifications will be also estimated using standardized dependent variables. The corresponding estimates indicate the change in SDs in the dependent variable over time. This facilitates the comparison to some previous results (Myers et al. 2019). However, some recent methodological papers (Baguley 2009; Pek and Flora 2018) suggest exercising caution in using standardized variables and refer to a number of unfavorable characteristics of standardized estimates such as being less robust and more prone to measurement error. Therefore, our reported baseline estimations will use unstandardized variables.

## Results

Descriptive statistics are included in Table 1. Descriptive statistics are computed using the weights provided by Eurofound. In this way, the numbers are representative of EU15 averages. They can be also considered as unconditional, raw averages of EU15 countries. However, for our regression analysis unweighted data will be used where country differences in terms of composition and data representativeness are taken into account by a wide range of explanatory variables.

**Table 1:** Descriptive statistics of covariates and work stress measures

Job strain *N*=74,959											
		1995		2000		2005		2010		2015	
		Mean (w)	*sd (w)*	Mean (w)	*sd (w)*	Mean (w)	*sd (w)*	Mean (w)	*sd (w)*	Mean (w)	*sd (w)*
Job strain		0.884	0.284	0.909	0.289	0.930	0.296	0.930	0.288	0.911	0.279
Psychological demands		1.411	0.318	1.425	0.309	1.452	0.311	1.448	0.299	1.452	0.301
Skill discretion		1.637	0.287	1.606	0.293	1.617	0.291	1.595	0.295	1.629	0.288
Decision authority		1.671	0.388	1.651	0.394	1.633	0.396	1.643	0.398	1.675	0.383

		% (w)	*N (uw)*	% (w)	*N (uw)*	% (w)	*N (uw)*	% (w)	*N (uw)*	% (w)	*N (uw)*
Gender	Male (%)	56.1%	6845	55.6%	8883	53.9%	5519	53.4%	8396	50.7%	8139
	Female (%)	43.9%	5526	44.4%	7961	46.1%	5974	46.6%	8952	49.3%	8764
Age	Below 30 (%)	30.1%	3749	28.2%	4938	25.7%	2635	24.3%	3843	21.2%	3056
	30–50 (%)	60.6%	7514	63.2%	10,604	63.5%	7379	63.4%	11,085	62.7%	10,834
	Over 50 (%)	9.3%	1108	8.6%	1302	10.8%	1479	12.3%	2420	16.1%	3013
Occupation	HC (high-skilled clerical)	34.1%	3774	34.2%	5224	37.3%	4259	39.3%	6480	38.5%	6376
	LC (low-skilled clerical)	29.6%	3839	30.3%	5730	28.3%	3530	28.7%	5754	31.5%	5451
	HM (high-skilled manual)	17.2%	2240	16.4%	2698	14.1%	1464	13.8%	2004	11.4%	1792
	LM (low-skilled manual)	19.1%	2518	19.1%	3192	20.3%	2240	18.2%	3110	18.6%	3284
Employment contract	Indefinite	81.3%	10,085	82.2%	13,684	78.4%	8957	80.1%	14,007	81.1%	13,550
	Fixed term	11.2%	1324	10.3%	1644	11.1%	1257	11.4%	1844	11.2%	1997
	Temp agency	3.4%	479	2.1%	326	1.9%	203	1.4%	310	1.6%	281
	Appr.	1.7%	198	1.8%	260	1.4%	118	1.3%	131	1.5%	145
	Other	2.4%	285	3.6%	930	7.2%	958	5.8%	1056	4.6%	930

Effort- reward imbalance *N*=45,399											
						2005		2010		2015	
						Mean (w)	*sd (w)*	Mean (w)	*sd (w)*	Mean (w)	*sd (w)*
ERI						0.891	0.234	0.891	0.227	0.889	0.229
Effort						1.453	0.311	1.449	0.299	1.455	0.300
Reward						1.657	0.183	1.652	0.176	1.665	0.189

						% (w)	*N (uw)*	% (w)	*N (uw)*	% (w)	*N (uw)*
Gender	Male (%)					53.9%	5538	53.6%	8375	50.9%	8045
	Female (%)					46.1%	5999	46.4%	8883	49.1%	8559
Age	Below 30 (%)					25.9%	2652	24.3%	3842	21.3%	3016
	30–50 (%)					63.3%	7406	63.6%	11,045	62.8%	10,680
	Over 50 (%)					10.8%	1479	12.2%	2371	15.9%	2908
Occupation	HC (high-skilled clerical)					37.3%	4282	39.4%	6462	38.8%	6316
	LC (low-skilled clerical)					28.4%	3552	28.7%	5731	31.4%	5344
	HM (high-skilled manual)					14.1%	1467	13.8%	2004	11.5%	1768
	LM (low-skilled manual)					20.2%	2236	18.0%	3061	18.3%	3176
Employment contract	Indefinite					78.4%	8992	80.2%	13,941	81.5%	13,383
	Fixed term					11.2%	1263	11.3%	1835	11.1%	1972
	Temp					1.9%	205	1.4%	305	1.6%	275
	Appr.					1.4%	116	1.3%	131	1.5%	145
	Other					7.2%	961	5.8%	1046	4.3%	829

Data presented as % (N) for categorical variables and mean (sd) for continuous variables. (w): weighted, (uw): unweighted; weights provided by EWCS. Psychological demands, skill discretion and decision authority are standardized and lie between 1 and 2; job strain lies between 0.5 and 2. Higher values correspond to higher psych. demands/skill disc/dec auth/job strain. Effort and reward are standardized and lie between 1 and 2; ERI lies between 0.5 and 2. Higher values correspond to higher effort/reward/ERI

The percentage of 30–50 year old employees is relatively stable during 1995–2015, while the percentage of the young is decreasing and the older is increasing. This is in line with ageing trends and the increases of statutory retirement ages in all countries. Regarding the distribution between occupational groups, the percentage of manual occupations is decreasing from 1995 to 2015, while the proportion of clericals is on an increasing trend.

The descriptive statistics are indicative of an increase in psychological demands and job strain between the years 1995 to 2005. Average scores of skill discretion and decision authority are decreasing slightly until 2010 and then bounce back. Short-term trends summarized by indicators of the effort-reward imbalance model do not indicate large changes in ERI. Average values of effort and reward suggest a U-shape profile where the 2010-values are likely to be influenced by the financial crisis.

### General model

Table 2 includes the full set of regression results based on the sample of EU15 countries. Estimated coefficients of the year dummies can be readily interpreted as changes in work stressors compared to year 2005. Accordingly, most of the change took place between 1995 and 2005. Movements in the work stressors from 2005 to 2015 have been small and mostly statistically insignificant. The results indicate an increasing trend in job strain from 1995 to 2005, which can be mostly explained by increases in psychological demands. The magnitude of the increase in job strain was 0.045 (on a scale from 0.5 to 2), and the change in psychological demands 0.060 (having a scale from 1 to 2) from 1995 to 2005. The corresponding standardized effect sizes are 0.159 SD for job strain and 0.196 SD for psychological demands (standardized results not shown). Changes of the control dimension as operationalized by skill discretion and decision authority have been small and mostly insignificant throughout the whole period. The analysis of single items underlying the job strain measure suggests that jobs have become more complex from 1995 to 2015, and more monotone from 2005 to 2015 (results not shown).

**Table 2:** Regression coefficients based on multilevel linear regressions analyzing the association between covariates and work stressors

		Job strain	Psych. demand	Skill disc.	Decision auth.	ERI	Effort	Reward
Year (ref. 2005)	1995	− 0.045	− 0.060	− 0.003	0.024			
		*(0.000)*	*(0.000)*	*(0.742)*	*(0.059)*			
	2000	− 0.010	− 0.040	− 0.033	− 0.012			
		*(0.347)*	*(0.004)*	*(0.000)*	*(0.329)*			
	2010	0.006	− 0.011	− 0.031	− 0.009	− 0.002	− 0.010	− 0.009
		*(0.578)*	*(0.446)*	*(0.001)*	*(0.478)*	*(0.858)*	*(0.454)*	*(0.033)*
	2015	0.005	0.004	− 0.008	0.001	0.001	0.006	0.009
		*(0.626)*	*(0.798)*	*(0.360)*	*(0.936)*	*(0.922)*	*(0.644)*	*(0.027)*
Gender (ref. male)	Female	0.023	− 0.001	− 0.059	− 0.017	0.011	− 0.000	− 0.020
		*(0.000)*	*(0.642)*	*(0.000)*	*(0.000)*	*(0.000)*	*(0.904)*	*(0.000)*
Age (ref. ≤30)	30<age<55	− 0.021	− 0.010	0.019	0.033	0.003	− 0.012	− 0.018
		*(0.000)*	*(0.000)*	*(0.000)*	*(0.000)*	*(0.326)*	*(0.001)*	*(0.000)*
	Age≥55	− 0.051	− 0.059	0.014	0.037	− 0.021	− 0.058	− 0.027
		*(0.000)*	*(0.000)*	*(0.000)*	*(0.000)*	*(0.000)*	*(0.000)*	*(0.000)*
ISCO (ref. HC)	LC	0.063	− 0.020	− 0.148	− 0.116	0.013	− 0.017	− 0.043
		*(0.000)*	*(0.000)*	*(0.000)*	*(0.000)*	*(0.000)*	*(0.000)*	*(0.000)*
	HM	0.115	0.032	− 0.173	− 0.151	0.054	0.038	− 0.055
		*(0.000)*	*(0.000)*	*(0.000)*	*(0.000)*	*(0.000)*	*(0.000)*	*(0.000)*
	LM	0.172	0.010	− 0.299	− 0.224	0.069	0.016	− 0.099
		*(0.000)*	*(0.003)*	*(0.000)*	*(0.000)*	*(0.000)*	*(0.000)*	*(0.000)*
Contract (ref. indefinite contract)	Fixed term contract	0.033	0.000	− 0.029	− 0.059	0.043	0.003	− 0.064
		*(0.000)*	*(0.938)*	*(0.000)*	*(0.000)*	*(0.000)*	*(0.504)*	*(0.000)*
	Temporary agency	0.080	0.016	− 0.080	− 0.104	0.101	0.051	− 0.106
		*(0.000)*	*(0.036)*	*(0.000)*	*(0.000)*	*(0.000)*	*(0.000)*	*(0.000)*
	Apprenticeship	− 0.019	− 0.035	0.041	− 0.080	− 0.039	− 0.038	0.028
		*(0.043)*	*(0.001)*	*(0.000)*	*(0.000)*	*(0.001)*	*(0.012)*	*(0.002)*
	Other	− 0.003	− 0.030	− 0.054	0.005	0.027	− 0.025	− 0.068
		*(0.530)*	*(0.000)*	*(0.000)*	*(0.425)*	*(0.000)*	*(0.000)*	*(0.000)*
NACE (ref. agriculture)	industry	0.041	0.075	0.037	− 0.019	0.048	0.081	− 0.001
		*(0.000)*	*(0.000)*	*(0.000)*	*(0.076)*	*(0.000)*	*(0.000)*	*(0.889)*
	services	0.025	0.054	0.012	− 0.006	0.043	0.062	− 0.011
		*(0.001)*	*(0.000)*	*(0.099)*	*(0.600)*	*(0.000)*	*(0.000)*	*(0.084)*
	Public admin.	− 0.050	− 0.022	0.060	0.029	− 0.025	− 0.017	0.019
		*(0.000)*	*(0.018)*	*(0.000)*	*(0.011)*	*(0.007)*	*(0.175)*	*(0.007)*
	Other services	− 0.048	− 0.021	0.044	0.036	− 0.007	− 0.010	− 0.006
		*(0.000)*	*(0.015)*	*(0.000)*	*(0.001)*	*(0.429)*	*(0.387)*	*(0.336)*
Constant		0.850	1.443	1.752	1.751	0.827	1.431	1.747
		*(0.000)*	*(0.000)*	*(0.000)*	*(0.000)*	*(0.000)*	*(0.000)*	*(0.000)*
Variance	Level 3 (Country)	0.002	0.002	0.003	0.005	0.001	0.002	0.001
		*(0.000)*	*(0.000)*	*(0.000)*	*(0.000)*	*(0.000)*	*(0.000)*	*(0.000)*
	Level 2 (Country years)	0.001	0.001	0.001	0.001	0.001	0.001	0.000
		*(0.000)*	*(0.000)*	*(0.000)*	*(0.000)*	*(0.000)*	*(0.000)*	*(0.000)*
	Level 1 (Individual)	0.071	0.089	0.065	0.134	0.049	0.088	0.029
		*(0.000)*	*(0.000)*	*(0.000)*	*(0.000)*	*(0.000)*	*(0.000)*	*(0.000)*
*N*		74,959				45,399		
*N* (groups)		15						

Regression coefficients based on multilevel regressions with three levels (level 1: individual, level 2: country-years, level 3: country). Sample: EU15, Waves included: 1995, 2000, 2005, 2010, and 2015 for job strain, psychological demands, skill discretion and decision authority. Waves included: 2005, 2010, and 2015 for ERI, effort, reward

Regarding the influence of individual covariates, the results indicate that women have significantly lower control (skill discretion and decision authority) and higher job strain compared to men. Psychological demands tend to decrease, while skill discretion and decision authority increase with age resulting in lower job strain among older employees compared to the younger ones. Workers in higher-skilled occupations have lower level of work stress compared to the lower-skilled, and employees with indefinite contract seem to be in more advantageous position in terms of job strain compared to those with fixed-term contract or temporary unemployment.

The trend-analysis of ERI is based on the waves 2005, 2010, and 2015. 2005 is used as the reference year. The impact of the financial crisis is visible on most estimates. Both effort and reward has a U-shape profile over 2005–2015 with a drop in 2010. Changes in ERI are statistically insignificant compared to 2005; however, the movement from 2010 to 2015 is statistically significant and indicates an improvement, which is mostly due to positive change in reward from 2010 to 2015. The relationship of individual covariates (gender, age, occupation, contract type) to ERI is qualitatively the same as in the case of job strain.

### Analysis by occupational groups

**Fig. 1:**
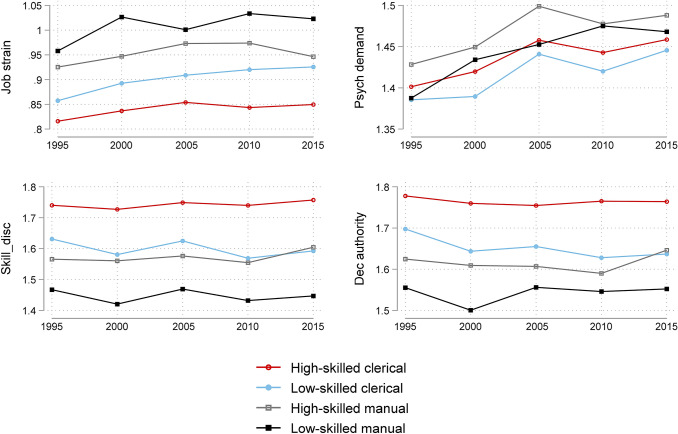
Predicted values of work stressors by occupational group. Work stressors based on demand-control model. Computation based on three-level multilevel regressions as specified in Table 3

**Fig. 2:**
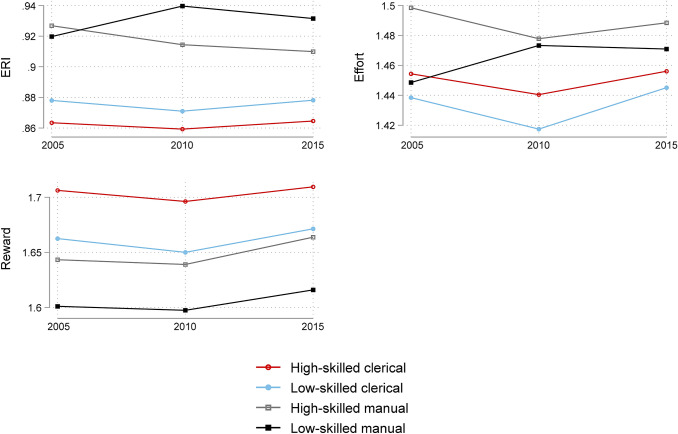
Predicted values of work stressors by occupational group. Work stressors based on ERI model. Computation based on three-level multilevel regressions as specified in Table 3

In the next step, the general model is expanded by a product term of occupational and wave dummies focusing the analysis on occupational differences. Results are summarized by Table 3 and a further visualization of the findings is provided by Figs. 1 and 2. Wald statistic indicated that the interaction terms were jointly significant.

**Table 3:** Predicted values of work stressors by occupation based on linear multilevel models

	1995	2000	2005	2010	2015	AME 2005 vs. 1995 (*p*-value)	AME 2015 vs. 2005 (*p*-value)	AME 2015 vs. 1995 (*p*-value)
Job strain (*N*=74,959)								
High-skilled clerical	0.816	0.837	0.854	0.843	0.850	0.038	− 0.004	0.034
	*(0.790*–*0.842)*	*(0.811*–*0.862)*	*(0.828*–*0.880)*	*(0.818*–*0.869)*	*(0.824*–*0.875)*	*(0.001)*	*(0.710)*	*(0.003)*
Low-skilled clerical	0.857	0.893	0.909	0.920	0.926	0.052	0.017	0.068
	*(0.831*–*0.883)*	*(0.867*–*0.918)*	*(0.883*–*0.935)*	*(0.895*–*0.946)*	*(0.900*–*0.951)*	*(0.000)*	*(0.149)*	*(0.000)*
High-skilled manual	0.925	0.947	0.973	0.974	0.947	0.048	− 0.026	0.021
	*(0.898*–*0.952)*	*(0.921*–*0.974)*	*(0.945*–*1.001)*	*(0.947*–*1.001)*	*(0.919*–*0.974)*	*(0.000)*	*(0.056)*	*(0.109)*
Low-skilled manual	0.958	1.027	1.001	1.034	1.023	0.043	0.022	0.065
	*(0.931*–*0.985)*	*(1.000*–*1.053)*	*(0.974*–*1.028)*	*(1.007*–*1.060)*	*(0.997*–*1.049)*	*(0.001)*	*(0.080)*	*(0.000)*
AME LM vs. HC (*p*-value)	0.142	0.190	0.147	0.190	0.173			0.031
	*(0.000)*	*(0.000)*	*(0.000)*	*(0.000)*	*(0.000)*			*(0.001)*

Psychological demands								
High-skilled clerical	1.401	1.420	1.458	1.443	1.459	0.056	0.001	0.057
	*(1.369*–*1.434)*	*(1.387*–*1.452)*	*(1.425*–*1.490)*	*(1.411*–*1.475)*	*(1.426*–*1.491)*	*(0.000)*	*(0.956)*	*(0.000)*
Low-skilled clerical	1.385	1.390	1.441	1.420	1.446	0.055	0.005	0.060
	*(1.353*–*1.418)*	*(1.357*–*1.422)*	*(1.408*–*1.474)*	*(1.388*–*1.452)*	*(1.413*–*1.478)*	*(0.000)*	*(0.757)*	*(0.000)*
High-skilled manual	1.428	1.449	1.499	1.478	1.488	0.071	− 0.011	0.060
	*(1.395*–*1.462)*	*(1.416*–*1.483)*	*(1.464*–*1.534)*	*(1.444*–*1.512)*	*(1.454*–*1.522)*	*(0.000)*	*(0.522)*	*(0.000)*
Low-skilled manual	1.387	1.434	1.453	1.475	1.468	0.065	0.016	0.081
	*(1.354*–*1.421)*	*(1.401*–*1.467)*	*(1.419*–*1.486)*	*(1.442*–*1.508)*	*(1.435*–*1.501)*	*(0.000)*	*(0.327)*	*(0.000)*
AME LM vs. HC (*p*-value)	− 0.0139	0.0143	− 0.00520	0.0323	0.00952			0.023
	*(0.074)*	*(0.036)*	*(0.517)*	*(0.000)*	*(0.145)*			*(0.020)*

Skill discretion								
High-skilled clerical	1.740	1.727	1.749	1.740	1.757	0.009	0.008	0.017
	*(1.709*–*1.772)*	*(1.696*–*1.758)*	*(1.718*–*1.780)*	*(1.709*–*1.771)*	*(1.726*–*1.788)*	*(0.399)*	*(0.391)*	*(0.087)*
Low-skilled clerical	1.631	1.581	1.625	1.569	1.592	− 0.006	− 0.032	− 0.039
	*(1.600*–*1.662)*	*(1.550*–*1.612)*	*(1.593*–*1.656)*	*(1.538*–*1.600)*	*(1.561*–*1.624)*	*(0.554)*	*(0.001)*	*(0.000)*
High-skilled manual	1.566	1.560	1.576	1.555	1.604	0.010	0.028	0.039
	*(1.534*–*1.598)*	*(1.529*–*1.592)*	*(1.543*–*1.609)*	*(1.522*–*1.587)*	*(1.572*–*1.637)*	*(0.386)*	*(0.023)*	*(0.001)*
Low-skilled manual	1.467	1.421	1.469	1.432	1.447	0.002	− 0.022	− 0.020
	*(1.435*–*1.499)*	*(1.389*–*1.452)*	*(1.437*–*1.501)*	*(1.400*–*1.463)*	*(1.415*–*1.479)*	*(0.852)*	*(0.044)*	*(0.065)*
AME LM vs. HC (*p*-value)	− 0.273	− 0.307	− 0.280	− 0.308	− 0.310			− 0.037
	*(0.000)*	*(0.000)*	*(0.000)*	*(0.000)*	*(0.000)*			*(0.000)*

Decision authority								
High-skilled clerical	1.778	1.760	1.755	1.765	1.764	− 0.023	0.009	− 0.014
	*(1.738*–*1.818)*	*(1.720*–*1.800)*	*(1.714*–*1.795)*	*(1.725*–*1.805)*	*(1.724*–*1.804)*	*(0.101)*	*(0.489)*	*(0.317)*
Low-skilled clerical	1.698	1.644	1.655	1.628	1.637	− 0.043	− 0.018	− 0.061
	*(1.657*–*1.738)*	*(1.604*–*1.683)*	*(1.615*–*1.696)*	*(1.588*–*1.668)*	*(1.597*–*1.677)*	*(0.00317)*	*(0.192)*	*(0.000)*
High-skilled manual	1.625	1.609	1.607	1.590	1.646	− 0.018	0.040	0.022
	*(1.583*–*1.666)*	*(1.568*–*1.651)*	*(1.564*–*1.650)*	*(1.548*–*1.632)*	*(1.604*–*1.689)*	*(0.295)*	*(0.0230)*	*(0.186)*
Low-skilled manual	1.555	1.501	1.556	1.546	1.552	0.001	− 0.004	− 0.003
	*(1.514*–*1.596)*	*(1.460*–*1.541)*	*(1.514*–*1.598)*	*(1.505*–*1.587)*	*(1.511*–*1.593)*	*(0.964)*	*(0.808)*	*(0.842)*
AME LM vs. HC (*p*-value)	− 0.223	− 0.259	− 0.199	− 0.219	− 0.212			0.011
	*(0.000)*	*(0.000)*	*(0.000)*	*(0.000)*	*(0.000)*			*(0.382)*

	2005	2010	2015	AME 2015 vs. 2010 (*p*-value)	AME 2015 vs. 2005 (*p*-value)
ERI (*N*=45,399)					
High-skilled clerical	0.863	0.859	0.865	0.005	0.001
	*(0.842*–*0.885)*	*(0.839*–*0.880)*	*(0.844*–*0.885)*	*(0.570)*	*(0.904)*
Low-skilled clerical	0.878	0.871	0.878	0.007	0.000
	*(0.857*–*0.899)*	*(0.850*–*0.892)*	*(0.857*–*0.899)*	*(0.449)*	*(0.989)*
High-skilled manual	0.927	0.914	0.910	− 0.005	− 0.017
	*(0.904*–*0.950)*	*(0.892*–*0.937)*	*(0.887*–*0.933)*	*(0.685)*	*(0.142)*
Low-skilled manual	0.920	0.940	0.932	− 0.008	0.012
	*(0.898*–*0.942)*	*(0.918*–*0.961)*	*(0.910*–*0.953)*	*(0.422)*	*(0.258)*
AME LM vs. HC (*p*-value)	0.056	0.080	0.067		
	*(0.000)*	*(0.000)*	*(0.000)*		

Effort					
High-skilled clerical	1.454	1.440	1.456	0.016	0.002
	*(1.425*–*1.484)*	*(1.411*–*1.470)*	*(1.427*–*1.485)*	*(0.249)*	*(0.903)*
Low-skilled clerical	1.438	1.417	1.445	0.028	0.007
	*(1.409*–*1.468)*	*(1.388*–*1.447)*	*(1.416*–*1.474)*	*(0.044)*	*(0.635)*
High-skilled manual	1.498	1.478	1.488	0.011	− 0.010
	*(1.466*–*1.531)*	*(1.446*–*1.509)*	*(1.457*–*1.520)*	*(0.502)*	*(0.539)*
Low-skilled manual	1.449	1.473	1.471	− 0.002	0.022
	*(1.418*–*1.479)*	*(1.443*–*1.503)*	*(1.441*–*1.501)*	*(0.871)*	*(0.135)*
AME LM vs. HC (*p*-value)	− 0.006	0.033	0.015		
	*(0.466)*	*(0.000)*	*(0.025)*		

Reward					
High-skilled clerical	1.706	1.696	1.709	0.013	0.003
	*(1.687*–*1.725)*	*(1.677*–*1.715)*	*(1.691*–*1.728)*	*(0.005)*	*(0.515)*
Low-skilled clerical	1.662	1.650	1.671	0.021	0.009
	*(1.643*–*1.682)*	*(1.631*–*1.669)*	*(1.652*–*1.690)*	*(0.000)*	*(0.086)*
High-skilled manual	1.643	1.639	1.664	0.025	0.020
	*(1.623*–*1.664)*	*(1.619*–*1.659)*	*(1.643*–*1.684)*	*(0.000)*	*(0.004)*
Low-skilled manual	1.601	1.597	1.616	0.018	0.015
	*(1.581*–*1.621)*	*(1.578*–*1.617)*	*(1.596*–*1.635)*	*(0.001)*	*(0.013)*
AME LM vs. HC (*p*-value)	− 0.105	− 0.099	− 0.094		
	*(0.000)*	*(0.000)*	*(0.000)*		

Predicted values based on multilevel modeling with three levels (level 1: individual, level 2: country-years, level 3: country). Covariates included in the regression: gender, age (< 30, 30–50, 50 <), contract type (indefinite, fixed term, temporary agency, apprenticeship, other), nace (5 groups), two-way interaction of wave dummies and occupation (4 groups). 95% confidence intervals in parenthesis. Average Marginal Effects (AME): *p*-values in parenthesis. Sample: EU15

The results highlight a social gradient in job strain and ERI increasing from high to low occupational classes. The social gradient is also visible in the control and the reward dimensions both increasing from low to high skill groups. This pattern is observed in each wave as illustrated by the last raw of each section in Table 3 presenting the AME of a change between the highest and lowest occupational groups (= difference between low-skilled manual (LM) and high-skilled clerical (HC) in the predicted values of work stressors in a specific wave). For instance, in 1995 job strain of high-skilled clericals is predicted to be 0.816 (on a scale from 0.5 to 2 where values close to and above 1 indicate high job strain), while the predicted value of job strain among low-skilled manuals is 0.958. The difference is 0.142 and significant at 1% level. Comparing these values (AME of a change between LM and HC) between the waves is indicative of widening or diminishing occupational inequalities in work stressors over time. Accordingly, a widening gap from 1995 to 2015 is observed for job strain, psychological demands and skill discretion.

The last three columns of Table 3 bring us to the analysis of trends by occupational position. Both job strain and psychological demands were significantly increasing within each occupational group from 1995 to 2015. The only exception is the high-skilled manual group where the improvement in skill discretion and decision authority from 2005 to 2015 compensated the rising psychological demands, which resulted in an insignificant increase in job strain from 1995 to 2015. The increase in both job strain and psychological demands were significantly larger in magnitude among the lowest skilled (LM) compared to the highest skilled (HC). The value of the difference in trends (from 1995 to 2015) between the HC and LM groups and the corresponding *p*-value is shown in the bottom right corner of each section of Table 3. For instance, in case of job strain the difference is 0.031 with a *p*-value of 0.001. As such, it indicates that the deterioration of working conditions from 1995 to 2015 was significantly larger in magnitude among the least skilled compared to the highest skilled. Analyzing trends in shorter periods suggests that most of the increase occurred from 1995 to 2005, while changes from 2005 to 2015 were moderate. Wave-to-wave movements in the control dimension were smaller and often insignificant. However, a few points are worth highlighting. From 1995 to 2015 skill discretion increased among the highest skilled and decreased among the lowest skilled. Though these changes were insignificant, the difference between the trends experienced by the highest and lowest skilled was significant suggesting a relative deterioration of working conditions among the least skilled. A second point worth emphasizing is that the position of high-skilled manuals (in terms of skill discretion) improved significantly from 2005 to 2015. Turning to the work stress measures based on the ERI model, the impact of the financial crisis is apparent indicated by a U-shaped profile of reward in each occupational group. The drop in rewards has been compensated from 2010 to 2015 in each occupational group. However, there are no significant movements in ERI or effort from 2005 to 2015.

In sum, the analysis indicated clear differences between the occupational groups with higher job strain in lower occupational classes. Job strain was increasing from 1995 to 2005 and the upward movement was driven by increases in psychological demands. This trend was found in each occupational group. Additionally, movements in some of the work stressors indicated a larger deterioration of working conditions from 1995 to 2015 among the least skilled than experienced by the highest skilled. Psychological demands increased more, skill discretion decreased resulting in a more pronounced deterioration of job strain from 1995 to 2015 among the least skilled compared to the highest skilled.

## Discussion

Our study used data from the last five waves of the EWCS and examined how psychosocial working conditions with relevance to health changed during the last 20 years. Working conditions were operationalized by the demand-control and the effort-reward imbalance models, and trends in composite work stress indicators were computed.

The main findings of our analysis indicate, *first*, an increasing long-term trend in job strain from 1995 to 2015, mostly driven by increases in psychological demands. Most of the change occurred from 1995 to 2005; changes from 2005 to 2015 were mostly insignificant. The magnitude of the change in job strain was 0.045 units on a scale of 0.5–2 corresponding to 0.159 SD change from 1995 to 2005. This was mainly driven by 0.060 units change in psychological demands (on a scale from 1 to 2) corresponding to 0.196 SD change in that variable from 1995 to 2005. Our findings indicate a larger change in job strain compared to Myers et al. (2019) documenting a 0.09 SD increase in job strain in the US from 2002 to 2014. Alternatively, the following thought experiment may also yield an illustration of the magnitude of the changes. The sample mean value of psychological demands/job strain in 1995 would increase by 0.071/0.044 units if all the responses on the survey item “Do you have to work to tight deadlines” were increased by one category upwards (e.g. respondents reporting “ ¾ of the time” were graded as “almost all of the time”, etc.).

*Second*, our results show clear differences between occupational groups, pointing to higher work stress in lower occupations. Though the prevalence of work stressors increased in each occupational group over the period of the study, the increase was significantly larger in magnitude among the least skilled than experienced by the highest skilled.

As previous studies focused on specific countries, shorter time periods and used a variety of different psychosocial work stress measures not being based on an underlying theory, it is difficult to draw comparison between those studies and ours. However, we can detect similarities with some papers. Our results are in line with previous literature pointing to increasing work demands from the beginning of the 1990’s (Greenan et al. 2014; Green and McIntosh 2001; Lopes et al. 2014). Using the EWCS, Green and McIntosh (2001) documented increasing work intensification between 1991 and 1995. Greenan et al. (2014) relying on the 1995, 2000 and 2005 waves of the EWCS pointed to a further intensification of work during that period. Myers et al. (2019) using US data found evidence of increasing job strain from 2002 to 2014. On the other hand, Gallie (2005) relying on Eurobarometer surveys from 1996 to 2001 did not find evidence of a general increasing trend in work pressure. The period from 2005 onwards is characterized by the impacts of the financial crisis. The results by Malard et al. (2013) using EWCS and a similar conceptualization of work stress can serve as a useful reference point. The authors, in line with our findings, found a significant decrease in skill discretion and decision latitude and a significant increase in job insecurity from 2005 to 2010. Regarding occupational differences in the prevalence of work stress, a social gradient between occupations has been already pointed out (Lunau et al. 2015; Wahrendorf et al. 2012; Myers et al. 2019). Our results provide further evidence suggesting persistent differences between occupations during the 20-year period of the study. Our additional results on the differing trends by occupation implying a larger deterioration of working conditions among the lowest skilled is in line with the findings of Malard et al. (2013) and Lopes et al. (2014). However, Myers et al. (2019) did not detect differential trends by occupation in the US from 2002 to 2014.

The period we have investigated is unique as it covers an exceptionally long period of 20 years including those years when the influence of globalization and the restructuring of work organizations were intensive. For instance, competitive pressure has been intensified, information and communication technology has turned to be an integral part of working life, work arrangements have become more flexible. These developments may suggest an intensification of work stress, especially among the lower skilled. Our results corroborate the expectations and confirm an intensification of work stress from 1995 to 2005. From 2005 onwards, the impacts of the financial crisis and the subsequent recovery are dominant.

Our results direct the attention to the vulnerable position of the least skilled both in terms of increasing psychological demands and decreasing skill discretion. Note that the group of the least skilled includes plant and machine operators (major group 8) and elementary occupations (major group 9). These groups might be disproportionately affected by occupational polarization induced either e.g. by technology, demographic developments or changes in life style. One way to stop the degradation of these jobs would be to enhance an occupational upgrading process, which would ultimately encourage employers to implement production techniques suited to better skilled workers – a solution suggested by the report of the European Centre for the Development of Vocational Training (Cedefop 2011). The use of various training schemes could be a tool towards this aim.

This study has several limitations. *First*, the original questionnaire items underlying the demand-control and ERI model are not all available in the EWCS. Thus, we created our work stress measures as close as possible to proxy the original constructs. Similar procedure was applied in previous work by Malard et al. (2013) and Niedhammer et al. (2012) both using the EWCS. The use of proxy measures is also encouraged by previous methodological studies pointing to close correspondence between partial or proxy measures and the validated scales (Fransson et al. 2012; Karasek et al. 2007). Some of our composite measures (skill discretion, reward) have a low internal consistency (Cronbach’s alpha less than 0.5). Low Cronbach’s alpha may be also the result of few survey items, or items not being unidimensional. Variables with low internal consistency may lead to the imprecision of the estimates. However, we found it important to include the chosen items as they represent closest the underlying theoretical constructs. Similarly low level of Cronbach’s alpha was also reported for some indicators by previous studies using the same database and a similar operationalization (Malard et al. 2013). Furthermore, we checked the criterion validity of job strain and ERI using logistic regressions to test the associations between these two stressors and self-reported health. The results showed significant associations between them. *Second*, our analysis might be biased due to sample selection. As the survey sample is composed of employees, we do not observe the working conditions of those who dropped out of the sample, due to e.g. disadvantageous working conditions. This may bias both the estimated level and trend of work stressors. Additionally, response rates are country-specific, and may change over time, which might also have an impact on the results. This selective nonresponse might be a problem if there are systematic differences between respondents and nonrespondents in terms of unobservable characteristics related to their work stress perceptions. To check whether country-specific response rates could affect our results, we analyzed the correlation between the response rate and the psychosocial work stressors, and found no strong associations in any case. *Third*, our analysis is based on a sample of only 15 countries, which might limit the generalizability of our results.

Despite these limitations, the present paper has several contributions to the existing literature. Our study is the first that uses five waves of the EWCS and analyzes long-term trends in work stressors over a 20-year period. Additionally, we carry out our analysis using proxies of validated measures of work stress, which have been previously shown to be related to detrimental health outcomes. Previous studies using similar operationalization of work stress focused only on two waves (Malard et al. 2013) or used more waves but relied on a different conceptualization of work stress (e.g. Greenan et al. 2014; Lopes et al. 2014). Importantly, our exhaustive analysis of trends in work stressors can help to identify vulnerable groups being most affected by unfavorable changes in labor markets and help our understanding of the mechanisms behind. In the present paper least skilled employees were found to be in the most disadvantageous position being characterized by high levels of psychological demands/effort, low levels of control and reward resulting in an elevated amount of work stress. Our results give rise to further research questions relating to the role of national policies and whether occupational differences can be buffered to some extent by the instruments of national policies. Important differences between countries in the prevalence of adverse psychosocial working conditions have already been pointed out by numerous papers (Malard et al. 2013; Niedhammer et al. 2012; Greenan et al. 2014; Lunau et al. 2013; Dragano et al. 2011). Active and passive labor market programs including different training schemes could be examples of such policies. We leave this question for a future research topic. Our current results point to the vulnerable position of the least skilled and call the attention for national policies being able to counteract some of the negative consequences of global changes.

## Policy conclusions

Our analysis has important policy relevance by highlighting the unfavorable position of the least skilled employees in terms of work stress. By identifying vulnerable groups in terms of exposure to work stress, our results contribute to developing more effective prevention measures and draw attention to the possible role of labor market policies. Reducing the prevalence of work-stress related sickness might not only contribute to decreasing health care costs, but also prevent early exit from the labor force. Prevention of early exit is especially actual in light of the current demographic trends towards an ageing society. Ensuring appropriate working conditions might have an important role in helping an ageing workforce to work longer and to remain productive.

## Data Availability

The EWCS datasets are stored with the UK Data Service (UKDS) in Essex, UK and are publicly available via their website (https://ukdataservice.ac.uk/). Users are required to be registered with the UK Data Service. Users who register have to accept the End User Licence (EUL) which is agreed to during the registration process. Computations were carried out using Stata version 14.1 licensed to UKD.

## Appendix

See Table 4.

**Table 4:** List of underlying EWCS survey items

Work stress measure	EWCS item	Response categories
Job strain (Psychological demands/Control)		
Psychological demands	Does your work include working at a very high speed?	1 (never) to 7 (all of the time)
	Does your work include working to tight deadlines?	1 (never) to 7 (all of the time)
Skill discretion	Does your job include learning new things?	1 (no) to 2 (yes)
	Does your job include solving complex tasks?	1 (no) to 2 (yes)
	Does your job include monotonous tasks	1 (yes) to 2 (no)
	Does your job include repetitive tasks of less than 10 min?	1 (yes) to 2 (no)
Decision authority	Are you able to choose the order of the tasks?	1 (no) to 2 (yes)
	Are you able to choose the methods of your work?	1 (no) to 2 (yes)
	Are you able to choose the rate or speed of your work?	1 (no) to 2 (yes)
ERI (effort/reward)		
Effort	Does your work include working at a very high speed?	1 (never) to 7 (all of the time)
	Does your work include working to tight deadlines?	1 (never) to 7 (all of the time)
Reward	I might loose my job in the next 6 months	1 (strongly agree) to 5 (strongly disagree)
	My job offers good prospects for career advancement	1 (strongly disagree) to 5 (strongly agree)
	Your job gives you the feeling of work well-done	1 (never) to 5 (always)
	Considering all my efforts and achievements in my job, I feel I get paid appropriately	1 (strongly disagree) to 5 (strongly agree)
	Your colleagues and/or manager support you	1 (none) to 2 (at least colleagues or manager)

Following Niedhammer et al. (2012) survey items were standardized in the following way:

$$item\_st_{i} = 1 + \frac{{\left( {item\_orig_{i} - 1} \right)}}{x - 1}$$

where *x* refers to the number of response categories as shown in Table 4.

## Abbreviations

ERI: Effort-reward imbalance
EWCS: European Working Conditions Survey
ISCO: International Standard Classification of Occupations
NACE: Nomenclature statistique des activités économiques dans la Communauté européenne
HC: High-skilled clerical
LC: Low-skilled clerical
HM: High-skilled manual
LM: Low-skilled manual
